# Reproductive timing and intensity in a Galápagos intertidal mollusc are modulated by thermal phases

**DOI:** 10.1038/s41598-025-06074-x

**Published:** 2025-07-01

**Authors:** Camila Gallardo-Duran, Cristina Vintimilla-Palacios, Isis Laura Alvarez-Garcia, Quetzalli Yasu Abadia-Chanona, Omar Hernando Avila-Poveda, Margarita Brandt

**Affiliations:** 1 Colegio de Ciencias Biológicas y Ambientales (COCIBA), Universidad San Francisco de Quito (USFQ), Quito, Ecuador; 2 Galapagos Science Center (GSC), Universidad San Francisco de Quito (USFQ) & University of North Carolina at Chapell Hill (UNC), Puerto Baquerizo Moreno, Galápagos, Ecuador; 3 Facultad de Ciencias del Mar (FACIMAR), Universidad Autónoma de Sinaloa (UAS), Mazatlán, Sinaloa Mexico; 4 Proyecto Quitón del Pacífico Tropical Mexicano, Mazatlán, Sinaloa Mexico; 5 Programa de Investigadoras e Investigadores por México, Secretaría de Ciencia, Humanidades, Tecnología e Innovación (SECIHTI), Ciudad de México, México; 6 Instituto Biósfera, Universidad San Francisco de Quito (USFQ), Quito, Ecuador

**Keywords:** Broadcast-spawning, ENSO, Gonad developmental stages, Reproductive investment, Reproductive phenology, Size at sexual maturity, Sperm competition, Animal physiology, Climate-change ecology, Tropical ecology, Ecology, Physiology, Zoology

## Abstract

The decline in finfish fisheries has increased the harvesting of coastal invertebrates, particularly molluscs. To understand how the endemic Galápagos chiton *Radsia goodallii* withstands harvest pressure, its reproductive traits were assessed on San Cristóbal Island across three El Niño thermal phases. Reproductive timing, duration, and intensity were found to vary significantly across thermal conditions, with a distinct cycle and peak gonadal investment approximately every four months. Reproductive intensity was highest during the cooler El Niño phase, whereas the duration of gonad maturity extended during warmer periods. Shifts in timing were evident in the onset of reproductive activity across phases. A male-biased sexual asymmetry in gonadal investment, combined with a higher number of females, suggested low sperm competition and potentially influenced male reproductive effort. Larger individuals exhibited greater reproductive capacity, indicating size-related reproductive optimization. Although a tropical species, *R. goodallii* displayed reproductive patterns more typical of temperate species, likely shaped by the Galápagos’ unique oceanographic conditions. These findings improve understanding of the species’ reproductive strategy and offer practical management insights, such as setting minimum catch sizes to protect juveniles until maturity or enforcing seasonal closures during reproductive peaks to support sustainable harvesting.

## Introduction

Declining finfish catches have prompted many fishers to target new species, leading to a rapid global expansion of fisheries, particularly of molluscs, often without sufficient scientific oversight^1,2^. Along the American Pacific Coast, several species of chitons (Mollusca: Polyplacophora) are now commercially harvested, contributing to food security and local economies. Some examples include *Katharina tunicata* in Alaska^3^, *Chiton articulatus* in México^4–6^, *Mesotomura echinata* and *Enoplochiton niger* in Perú^7^, *Radsia goodallii* and *Radsia sulcatus* in the Galápagos Islands^8,9^, and several species in Chile, including *Chiton granosus, Chiton magnificus, Mesotomura echinata* and *Chaetopleura peruviana*^10–12^*.*

The Galápagos Islands hosts a total of 12 chiton species^13–15^, most of which are small and uncommon. However, two of these species, *Radsia goodallii* and *Radsia sulcatus* are larger and more abundant^13,16–18^, making them attractive to harvesting. *Radsia goodallii* can reach up to 160 mm in length^8^, while *R. sulcatus* reaches up to 120 mm^13^. Both species are endemic to the Galápagos, although *R. goodallii* has also been sporadically reported from Cocos Island in Costa Rica^19–21^, and from Santa Elena in mainland Ecuador^22^.

Locally known as “canchalaguas”, both *Radsia goodallii* and *Radsia sulcatus*, have a muscular foot, with mean weight/size around 5.4 ± 3.2 g and 5.5 ± 1.1 cm (n = 464, unpublished data), and is considered a delicacy. Historically harvested for subsistence, they are now part of a small-scale commercial fishery. Chitons are sold to local restaurants, hotels, and grocery stores^8,9,23^, and they are exported to mainland Ecuador^24^. Dishes in Galápagos cost between $6 and $20 USD, depending on the season^24,25^.

The chiton fishery in the Galápagos is regulated under several measures. First, harvesting is permitted only by hand in the intertidal zones of the four inhabited islands (Santa Cruz, San Cristóbal, Isabela and Floreana) under the “pesca peatonal modality”, which consists of harvesting benthic invertebrates while walking (without boats or fishing gear). Second, only licensed artisanal fishers who are permanent residents may participate, and third, exportation to the mainland is limited to 2 lbs × person⁻^1^ × day⁻^1^^26–29^. However, chitons can be harvested year-round^26,27^, and reported capture rates of approximately 18 ± 4 ind × h^−1^ for *R. goodallii* and 2 ind × h^−1^ for *R. sulcatus*^23,24,30^ raise concerns about long-term sustainability.

Given the fishing pressure on chitons, understanding their reproductive biology is critical for effective management. As ectotherms, chitons are influenced by environmental temperature, which affects reproductive timing and effort. This study investigated whether there is modulation in the intensity and duration of gonadal maturity in *Radsia goodallii*, and how this modulation varies across contrasting thermal phases associated with El Niño Southern Oscillation (ENSO) events. Specifically, variation in size structure and reproductive traits of *R. goodallii* was assessed on San Cristóbal Island during cooler (La Niña) and warmer (El Niño) phases. The focus was on the Reproductive Season, examining its intensity and duration through Maximum Gonad Investment, as well as Sexual Asymmetry in gonadal development using the Gonadosomatic Index. Additionally, the relationship between body size and reproductive investment in both sexes was explored. *R. goodallii* was selected as the focal species due to the relative scarcity of *R. sulcatus* on San Cristóbal Island, where the research facility, the Galápagos Scienter (GSC), is located.

## Materials and methods

### Sampling area

The Galápagos Islands have been a World Heritage Natural Site since 1978^31^. Its Marine Reserve is a renowned tourist destination^32,33^, a hub for artisanal fishing^34,35^, and a significant area for research. All these activities are governed by a Management Plan that employs a socio-ecological system framework^36^.

**Fig. 1:**
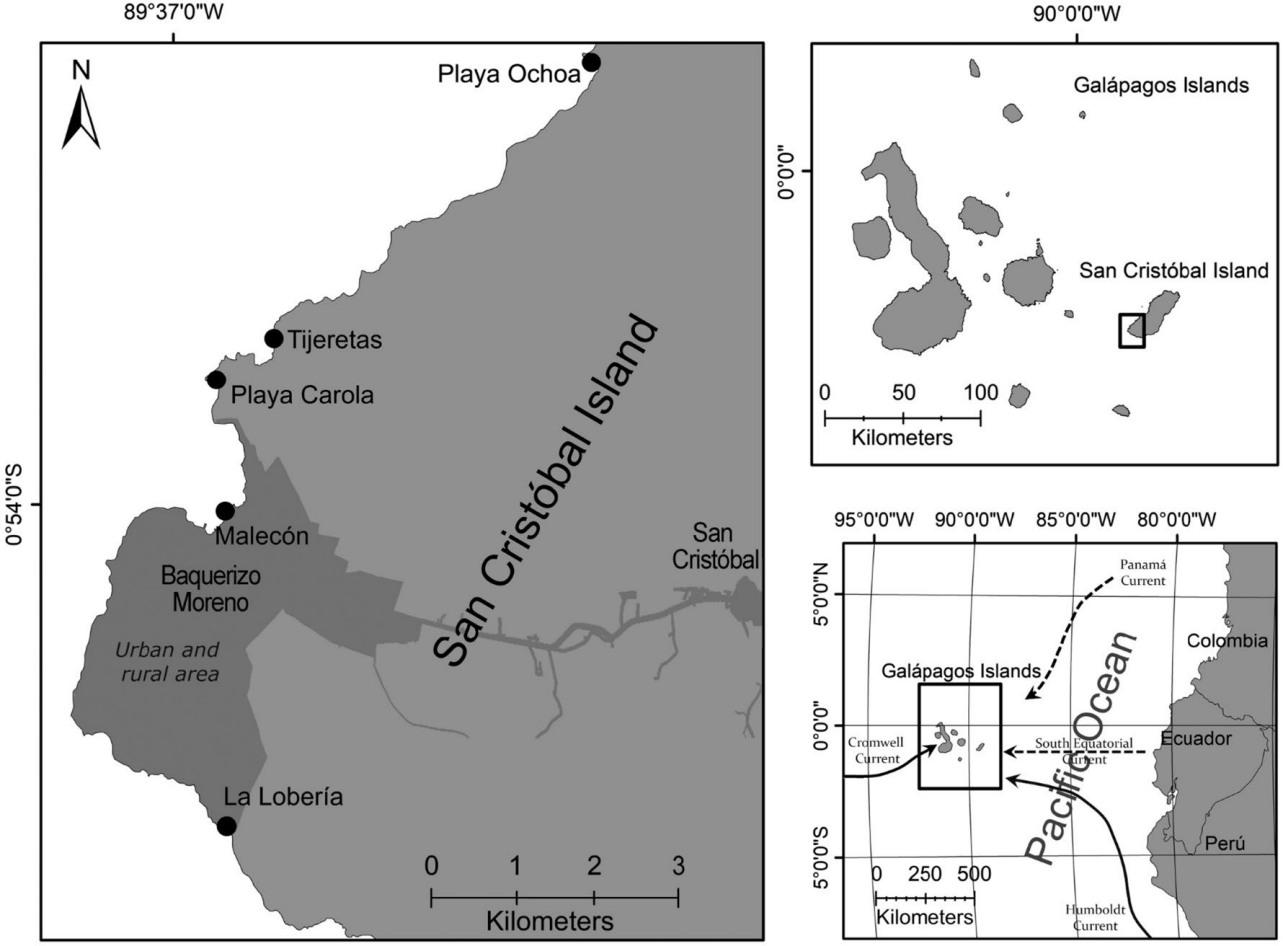
Sampling area and map of the collection sites of *Radsia goodallii* along the southwestern coast of San Cristóbal Island, Galápagos Archipelago, Ecuador. Dashed arrows in right bottom panel are warm currents, and solid arrows are cold currents.

Four oceanic currents influence the Galápagos Islands: (1) the South-Equatorial Current, a flow of tropical and subtropical waters moving from the east to the west across the archipelago; (2) the Humboldt Current, a nutrient-rich and cold water mass originating in the Southern Hemisphere, off the Coast of Perú; (3) the Equatorial Undercurrent, also known as the Cromwell Current, which flows from the west to the east, bringing nutrients and cold waters to the surface; and 4) the tropical and nutrient-poor Panamá Current, which originates off the coast of Central America and flows southwest, primarily influencing the northern part of the Galápagos Archipelago (Fig. 1). The Humboldt and the Panamá Currents exhibit seasonal variation, with the Humboldt Current dominating from June to December, and the Panamá Current prevailing from January to May^37,38^. These regional conditions, dictate the seasonal fluctuations and regulate the Sea Surface Temperature (SST) of the Galápagos Islands^39,40^. Additionally, as the Galápagos Archipelago lies within the Eastern Tropical Pacific (ETP) Ecoregion^41^, ENSO also plays a critical role in the regional oceanography, causing cooler or warmer than average SSTs and sea level anomalies related to the thermocline depth^42,43^. The cooler La Niña phase (known as the Equatorial Cold Tongue) leads to a rise in the cold thermocline and an increase in nutrient availability^44^, while the warmer El Niño phase reduces nutrients levels, intensifies wave action, and raises sea levels, which in turn, affect food availability across different food chain levels^45^.

### Sampling design and thermal phases

The collection of chiton samples took place in the rocky intertidal zone on the southwestern coast of San Cristóbal Island (0° 47′ 58.02″ S, 89° 23′ 55.15″ W; Fig. 1). This intertidal zone is characterized by a shallow platform fragmented by sandy beaches, sandbars, protected bays, cliffs, gravel bars and bedrock-floored ravines^46^. These features are interspersed with areas of exposed basaltic rock fragments^47,48^. The algal communities are dominated by crustose coralline and turf algae, while the animal communities are composed of marine iguanas, herbivore fishes, crustaceans, molluscs and echinoderms^45,49–51^.

Only adult chitons were collected to assess the reproductive characteristics of *Radsia goodallii*. Adult size was determined based on foraging behavior associated with the juvenile-adult transition, in which adults emerge above the rock surface, making them visible, whereas juveniles remain hidden^30^. These Adults exhibited widths ranging from 28 to 30 mm (measured along the left–right axis of the widest intermediate valve, including the mantle girdle), which corresponds to a Total Length (TL) between 47 and 65 mm^30^; therefore, chitons with a TL > 47 mm were considered adults.

Between February 2018 and February 2019 up to 31 adult individuals of *Radsia goodallii* were collected monthly. As *R. goodallii* exhibits a clumped distribution, typically with about a dozen individuals per clump (Brandt, *pers. obs.*), a maximum of four individuals were collected per clump to minimize the impact on the population. Chiton sampling was conducted along approximately 16 km of shoreline, between Playa Ochoa and La Lobería (Fig. 1). Chitons were collected during low tide and brought to the laboratory alive. To keep the chitons uncoiled for measurements and to preserve gonad anatomy for histology, they were relaxed, fixed, and preserved according to the protocols established by^52^.

Our sampling period covered three distinct ENSO thermal phases determined by SST anomalies, as indicated by the Oceanic Niño Index (ONI)^53^: (1) a La Niña cooler phase from February to April 2018, characterized by SST anomalies ≤ − 0.5 °C; (2) a neutral phase from May to August 2018, with SST values falling between − 0.5 and 0.5 °C; and (3) a El Niño warmer phase from September 2018 to February 2019, marked by SST anomalies ≥ 0.5 °C. These thermal phases do not correspond to the seasons explained in the previous section, as the proper the cool season runs from June to December and the warm season from January to May ^37,38^.

### Morphological measurements and gonad extraction

After preservation, the Scleritome Length (SL) of each chiton was measured using a caliper (±0.1 mm). SL is defined as the length of the assembled plates (excluding the mantle girdle) along the anteroposterior axis. To facilitate comparison with other studies, SL was converted to the measurement that includes the mantle girdle: the Total Length (TL). This conversion was performed using linear interpolation based on the TL and SL measurements of the chitons sampled in this study, resulting in the formula: TL = SL + 7 mm, which is consistent with that observed for the adult stage of *Chiton articulatus*^126^. The entire gonad was then extracted from each specimen following the method described by^54^, which accounts for the single sac-shaped gonad characteristic of Polyplacophorans^55^. Body and gonad weights were subsequently measured using a HAUSE analytical balance (± 0.001 g).

### Assignment of sex

*Radsia goodallii* does not exhibit sexual dimorphism; however, the gonad color differs between sexes, as has been documented in several other chiton species^56–60^. Therefore, the gonad color was used to determine the sex of each individual: testes exhibit shades of orange, reddish and salmon, while ovaries are olive green^8^, similar to those of *Chiton articulatus*^61^. Additionally, sex was confirmed through microscopic examination of histological sections.

### Assignment of the microscopic gonad developmental stages (GDSs)

Following a standard histological procedure and a modified^62^, trichrome stain (i.e., Groat’s hematoxylin, erythrosine B-Orange G, and trypan blue and/or light green), a microscopic gonadal examination protocol for chitons, as described by^54^, was employed. Each specimen was assigned to one of the five Gonad Developmental Stages (GDS): GDS-1 Goniogenesis (oogonial and spermatogonial proliferation), GDS-2 Development, GDS-3 Ripe, GDS-4 Spawning, and GDS-5 Resting. This classification was based on a panoramic examination of each transverse-section of the gonad^54^, which takes into account the anatomical structure of the single sac-shaped gonad of Polyplacophorans^52,54,63–66^.

The protocol developed by^54^ allows for the identification of tissue platelets, the presence of ciliated and non-ciliated blood vessels, the filling of the lumen with gametes, and the types of sex cells^59,63^. The panoramic procedure facilitates the observation of gamete displacement within the single sac-shaped gonad, from the ventral side toward the dorsal side^64^. This comprehensive approach minimizes the risk of inaccurate determinations of the reproductive cycle and season that could result from misclassifying the GDSs if only a portion of the Polyplacophoran gonad is examined^54^.

### Data analyses

#### Size Structure

To examine the size structure of the sampled chitons, TL frequency distributions were constructed separately for each sex with TL data grouped into 6-mm class intervals according to Sturges’ rule^67,68^ and descriptive statistics are presented. The normality of TL frequency distributions of was assessed using the Kolmogorov–Smirnov (K–S) test. When data deviated from normality, the nonparametric Mann–Whitney U (M-W-U) test was employed to evaluate significant differences in TLs data set between the two sexes.

#### Temporal variation in chiton length

Differences in total length (TL) between months and among thermal phases were tested separately for each sex. Prior to parametric analysis, the normality of TL data was assessed within each group using the Shapiro-Wilk test, and homogeneity of variances was tested using Levene’s test. When the assumptions of normality or homoscedasticity were violated, non-parametric methods were applied accordingly. Monthly TL data were analyzed using one-way ANOVA, followed by Fisher’s LSD test for post-hoc comparisons^69^. In contrast, TL differences across thermal phases were assessed using Kruskal–Wallis (K-W) one-way analysis of variance on ranks, followed by Dunn’s post-hoc test to account for unequal sample sizes^70^. All statistical analyses were conducted using STATISTICA 8, and statistical significance was accepted at *P* < 0.05.

#### Sex ratio

The males-to-females sex ratio was calculated monthly and annually. Deviations from a 1:1 ratio were tested with a Chi-square Goodness of Fit test, using Yate’s correction (*χ*^*2*^_*c*_) with 1 degree of freedom^70^.

#### Size at sexual maturity

The size at sexual maturity (expressed as *TL*_50%_) was calculated using the cumulative TL size frequencies (grouped into 6 mm class intervals) of organisms in the GDS-3 Ripe and GDS-4 Spawning stages. The *TL*_50%_ value was derived by fitting the data to a sigmoidal logistic function using the Curve Expert Professional 2.6.5 Software (http://www.curveexpert.net/):

$${TL}_{50\%}=\frac{a}{{1+b*e}^{(-cx)}}$$

where *a*, *b* and *c* are model parameters and *x* represents the TL of the chitons in the GDS-3 Ripe and GDS-4 Spawning stages.

#### Sexual asymmetry in the gonadosomatic index (GSI)

In broadcast-spawning marine invertebrates, the Maximum Gonad Investment (MGI or GSI_max_, see next section for details) can provide a reliable indication of the reproductive season at the population level. However, significant variation in the GSI_max_ may occur between sexes just before spawning, influenced by extrinsic factors such as latitude, local temperature regimes, somatic requirements, or food availability^71,72^. To minimize the effects of environmental factors, it is useful to compare the Relative Gonad Expenditure (RGES) between the sexes, as it is an intrinsic factor and informs on the sexual asymmetry in the GSI_max_^61,66,72,73^. This comparison also helps understand the selective forces of sperm competition and sperm limitation^72,74,75^. The RGES was calculated as the natural logarithm of male-to-female GSIs, following^61,72^. This index yields three possible outcomes: (1) if RGES > 0 or M > F, it is a male-biased asymmetry; (2) if RGES ≈ 0 or F = M, there is equal gonad expenditure between sexes; and (3) if RGES < 0 or F > M, it is a female-biased asymmetry. The standard deviation (*σ*) of the RGES ratio was estimated according to^76,77^ and the absolute values were transformed to natural logarithms for consistency with the RGES calculation^61,66^.

#### Reproductive cycle: frequencies of the gonad developmental stages (GDSs)

Given that the reproductive cycle depends on intrinsic and extrinsic factors^78^, each reproductive cycle was identified by the co-occurrence of all GDSs within the population at each month. Monthly GDS frequencies for each sex separately, and together, were calculated and represented as stacked bar graphs. The reproductive cycle was then divided into two periods^79^: The Reproductive readiness period (Rrp), which includes three of the five GDSs: GDS-5 Resting, GDS-1 Goniogenesis and GDS-2 Development; and the Reproductive achievement period (Rap), encompassing the GDS-3 Ripe and GDS-4 Spawning stages, representing the typical “reproductive season” for broadcast-spawning marine invertebrates.

#### Reproductive season: intensity and duration of the maximum gonad investment (MGI)

The MGI of an organism refers to the highest percentage value of the Gonadosomatic Index (GSI_max_)^61,66,72,73^. The GSI (expressed by gonad weight as a percentage of body weight devoted into reproduction) is calculated using the traditional formula: GSI = [GW/TW] × 100, where GW is the gonad weight and TW is the total body weight. The GSI_max_ percentage values, corresponding to a gonad full of ripe gametes (GDS-3, Ripe stage), represent the maximum intensity of the reproductive season, particularly in broadcast-spawning marine invertebrates. Lower GSI percentage values, indicating the emptying of the gonad, correspond to the GDS-4 Spawning or GDS-5 Resting stages^61,66,73,79^.

The duration of the reproductive season is defined as the group of months adjacent to the month with the GSI_max_ (MGI) percentage value that do not show significant differences ^66,73,79^. Since the GSI data are expressed as a percentage, they were normalized using an arcsine square root transformation^80^ before being tested with a One-way Analysis of Variance (ANOVA) to detect significant differences in GSI_max_ by sex, month, and thermal phase. This was followed by Fisher’s Least Significant Difference (LSD) post hoc tests for multiple comparisons. Statistical significance was set up at *p* < 0.05^69^. All statistical analyses were carried out using Statistica 8.0 ® and SigmaPlot 12 ®.

Since GSI is a function of gonad weight gained toward maturation, it is independent of each GDS in broadcast-spawning species^61,79,81–83^. Therefore, to assess population-level reproductive intensity for each GDS, the range of GSI values was measured by GDS, sex, and thermal phases. Additionally, to emphasize the intensity of the MGI, the GSI range for the GDS-3 Ripe stage was obtained by sex and thermal phase. The variation in reproductive intensity with body size in *Radsia goodallii* males and females across thermal phases was also examined to evaluate the influence of allometric relationships between the mature egg GSI or size and adult body size, as observed in other chitons belonging to the Family Chitonidae (see *Chiton granosus*, *Chiton cumingsii*, and *Chiton magnificus* in^60^, and *Chiton articulatus* in^66^.

### Ethical statement

We adhered to bioethical practices in the treatment of animals during research, considering the nociception and pain response of aquatic invertebrates^84^. This involved careful selection of specimen size, along with human methods for collection, relaxation, fixation, and preservation^52^. Our procedures aligned with the welfare and care standards for animals, applying the Five R´s Principle as closely as possible for invertebrates^85^, and were guided by the ethical principles of beneficence and nonmaleficence. This research was undertaken under permits PC-14-18 and PC-67-19 from the Galápagos National Park Directorate awarded to M. Brandt, guided by the ethical principles of autonomy and justice.

## Results

### Size structure

**Fig. 2:**
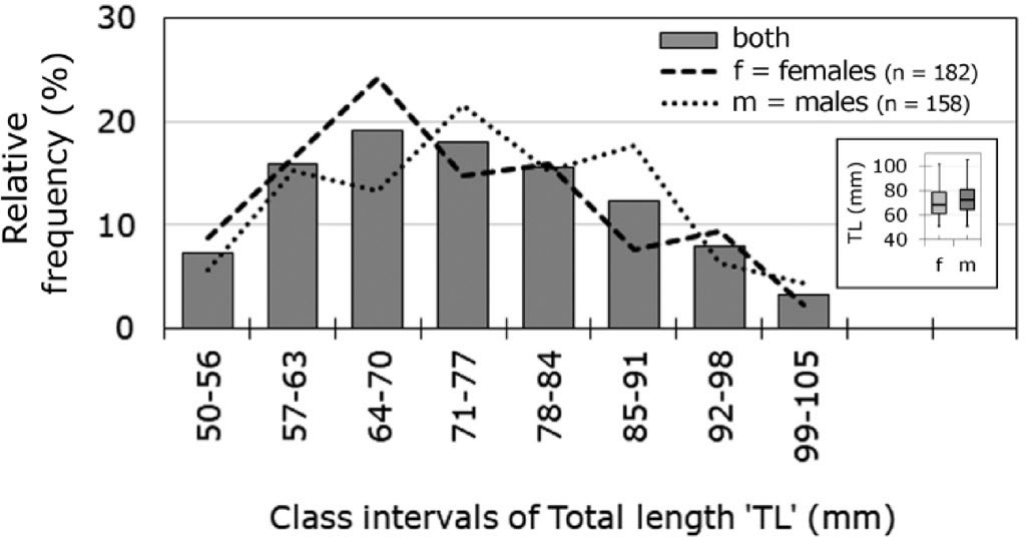
Size Structure of *Radsia goodallii* sampled in San Cristóbal Island, Galápagos Archipelago, Ecuador. Total Length (TL) frequency distributions are shown for females, males and all individuals combined, regardless of thermal phases. Inset shows median TL values.

In total, 340 adult *Radsia goodallii* individuals from San Cristóbal Island were measured, consisting of 182 females and 158 males, and the descriptive statistic for this data set are as follows. The mean TL for adult females was70.2 ± 11.4 mm with a size range of 51–101.5 mm; while the mean TL for adult males was 72.6 ± 11.2 mm with a size range of 51–105 mm (Fig. 2 inset). Furthermore, TL distribution of females had maximum frequency in the 64–70 mm class interval, whereas males showed a maximum frequency in the 71–77 mm class interval (Fig. 2), a size range that largely corresponds to the estimated size at sexual maturity (Fig. 4, see below).

Inspection of the normality of the TL distribution for each sex indicated a non-normal TL distribution for females, skewed towards smaller sizes (K-S test: D = 0.1104, *p* =  0.0001), whereas male TLs were relatively uniformly distributed (K-S test: D = 0.0484, *p* =  0.1077, Fig. 2). Median TL of females (68.13 mm) was significantly smaller than that of males (72.14 mm, M-W-U test: U = 124,490, *p* = 0.033, Fig. 2).

### Temporal variation in chiton length

**Fig. 3:**
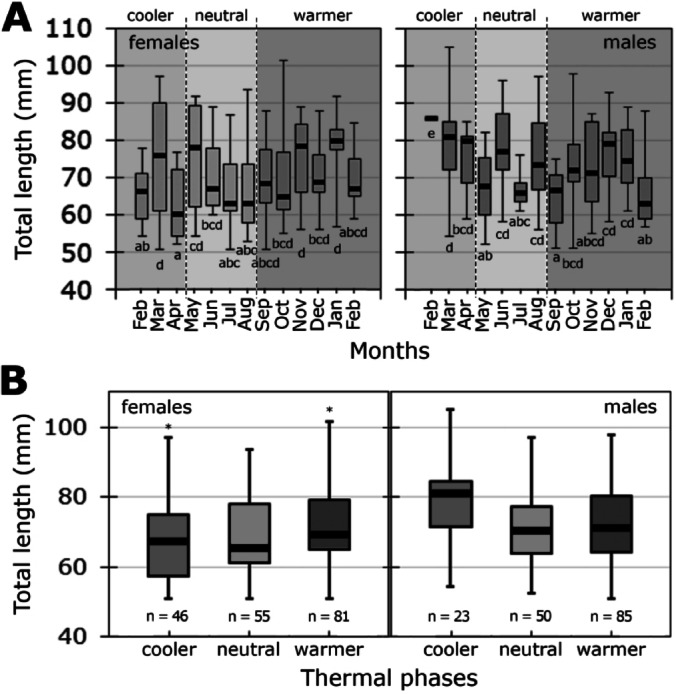
Temporal variation in Total Length (TL) of *Radsia goodallii* females and males across months (**A**) and thermal phases (**B**) on San Cristóbal Island, Galápagos Archipelago, Ecuador. Each thermal phase is highlighted with grey shading. Horizontal lines are medians ± quartile deviation (bold line and box) with minimum–maximum ranges as whiskers. Different letters indicate significant differences in median TLs between months at p < 0.05. In **B**) asterisks in females denote phases that showed differences between them, while males did not show significant differences among thermal phases (*p* = 0.1318). The *n* indicates the number of chitons recorded for each sex in each thermal phase during the entire sampling period.

Both female and male median TL values varied significantly across months (one-way ANOVA tests: females: F_12,169_ = 2.363, *p* = 0.008; males: F_12,145_ = 2.334, *p* = 0.009; Fig. 3A). When analyzed by thermal phase (Fig. 3B), females showed median TL differences between the cooler and the warmer phase, with a tendency toward larges sizes during the cooler phase (K-W test, H_2, N=182_ = 5.998, *p* = 0.0498). Males showed a similar trend, although the differences were not statistically significant (K-W test, H_2, N=158_ = 4.053, *p* = 0.1318).

### Sex ratio and size at sexual maturity

**Fig. 4:**
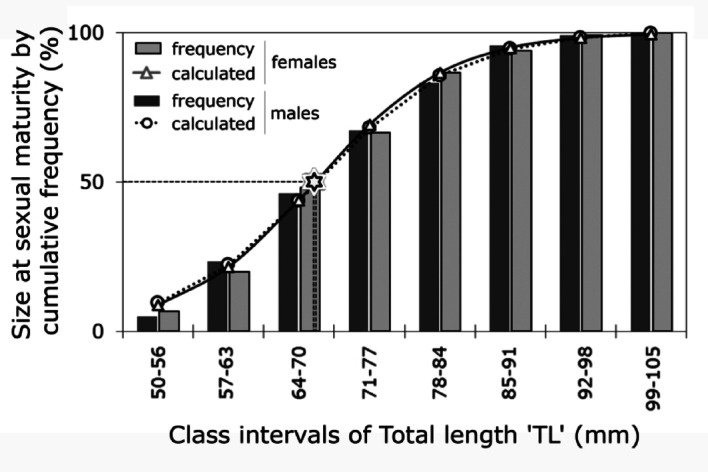
Size at Sexual Maturity (TL_50%_) for *Radsia goodallii* females and males at San Cristóbal Island, Galápagos Archipelago, Ecuador*.* Horizontal-dashed line represents 50% of adult chitons that were at GDS-3 Ripe and GDS-4 Spawning stages. Smooth lines (regular and dotted) indicate the calculated tendency of the sigmoidal model. Bars refer to the observed cumulative size-frequencies of each sex (light grey = females, dark grey = males). The star shows the TL_50%_ at about 69 mm.

The total of 158 males (46.5%) and 182 females (53.5%) collected throughout the duration of the study, represented an annual male-to-female ratio of 0.87:1, which did not deviate significantly from the expected 1:1 ratio (*X*^*2*^ = 13.31, *p* = 0.35, Table 1). However, the sex ratio significantly differed from a 1:1 expectation in February 2018, when females were more numerous (0.13:1 male-to-female ratio; *p* = 0.04; Table 1). On the other hand, the TL_50%_ at sexual maturity was 69.3 mm for males and 69.5 mm for females (Fig. 4), which corresponded to their maximum frequency in the 64–70 class interval (Fig. 2).

**Table 1:** Monthly and annual sex ratio for *Radsia goodallii* over the duration of this study (February 2018 to February 2019) on San Cristóbal Island, Galápagos Archipelago, Ecuador.

Thermal phase	Month	Nm	Nf	Nt	%m	%f	m:f	X^2^c	*P*-value
Cooler	Feb-18	2	15	17	11.76	88.24	0.13:1	4.24	0.04*
	Mar-18	13	15	28	46.43	53.57	0.87:1	0.02	0.89
	Apr-18	8	16	24	33.33	66.67	0.50:1	1.02	0.26
Neutral	May-18	19	12	31	61.29	38.71	1.58:1	0.58	0.38
	Jun-18	9	15	24	37.50	62.50	0.60:1	0.52	0.47
	Jul-18	6	13	19	31.58	68.42	0.46:1	0.95	0.27
	Aug-18	16	15	31	51.61	48.39	1.07:1	0.00	1.00
Warmer	Sept-18	12	15	27	44.44	55.56	0.80:1	0.07	0.79
	Oct-18	13	15	28	46.43	53.57	0.87:1	0.02	0.89
	Nov-18	12	14	26	46.15	53.85	0.86:1	0.02	0.89
	Dec-18	19	11	30	63.33	36.67	1.73:1	0.82	0.37
	Jan-19	16	8	24	66.67	33.33	2.00:1	1.02	0.31
	Feb-19	13	18	31	41.94	58.06	0.68:1	0.26	0.53
	Total	158	182	340	46.47	53.53	0.87:1	13.31	0.35

Nm, number of males; Nf, number of females; Nt, total number of males and females; %m, percentage of males; %f, percentage of females; m:f, male-to-female ratio; *X*^*2*^_*c*_, Chi-square Goodness of Fit with Yate’s correction for continuity only by month. Asterisk denotes deviation from the expected males-to-females 1:1 ratio and indicates a significant difference at *p* ˂ 0.05. Each thermal phase is highlighted with grey shading.

### Sexual asymmetry in the gonadosomatic index (GSI)

**Fig. 5:**
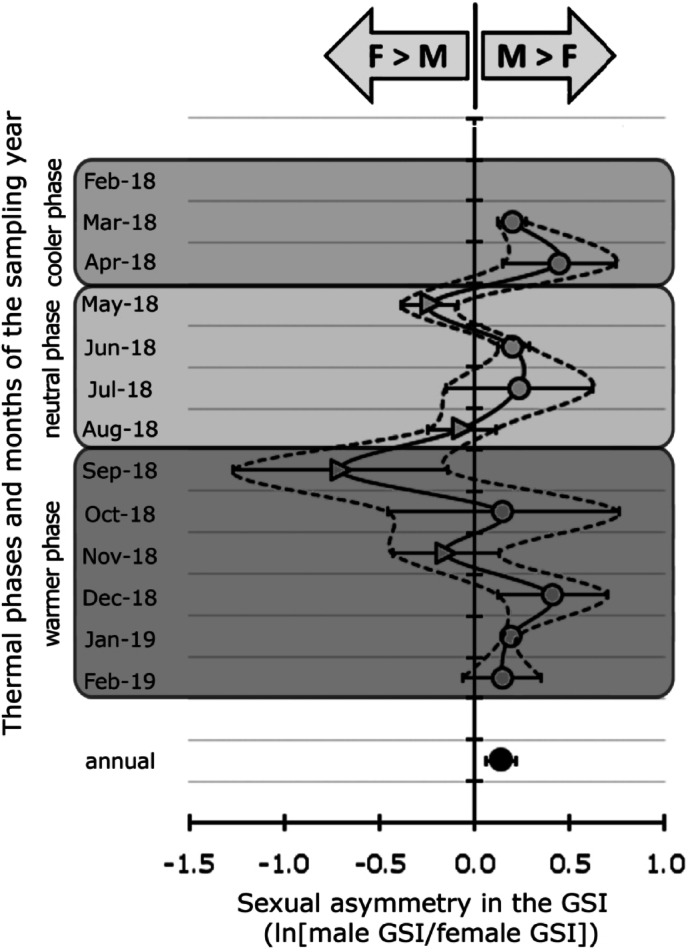
Sexual Asymmetry in the Gonadosomatic Index (GSI) shown as the variation in the Relative Gonad Expenditure of the Sexes RGES, (± SD) of *Radsia goodallii* from San Cristóbal Island, Galápagos Archipelago, Ecuador. The RGES values include all Gonad Developmental Stages (GDSs 1 to 5) for each sex. F > M refers to female-biased RGES asymmetry (triangular symbols), and M > F to male-biased RGES asymmetry (circular symbols). Each thermal phase is highlighted with grey shading.

Based on the relative gonad expenditure, *Radsia goodallii* exhibited no symmetry between sexes (Fig. 5). Male-biased asymmetry (M > F, RGES > 0) prevailed most of the time, ranging from 0.14 to 0.45. Female-biased asymmetry (F > M, RGES < 0) was observed in May, August and November, and maximum at − 0.70 in September. The overall annual RGES showed a slight male-biased asymmetry of 0.14 (Fig. 5, bottom panel).

### Reproductive cycle: frequencies of the gonad developmental stages (GDSs)

**Fig. 6:**
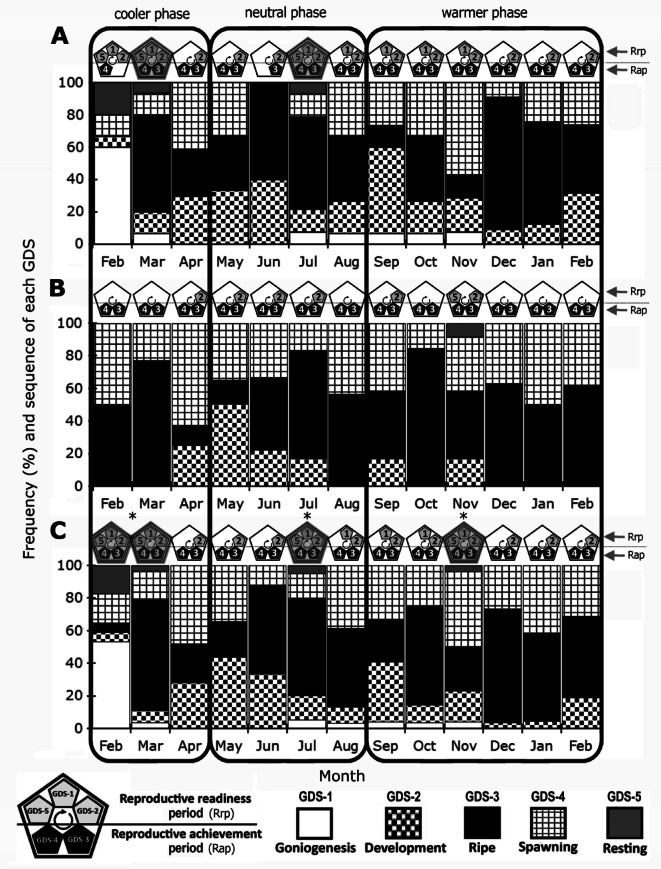
Monthly Frequencies of *Radsia goodallii* Gonad Development Stages (GDSs) across thermal phases from February 2018 to February 2019 at San Cristóbal Island, Galápagos Archipelago, Ecuador. (**A**) females, (**B**) males, (**C**) both sexes. Periods of the Reproductive Cycle (Rrp = Reproductive readiness period, Rap = Reproductive achievement period, see Avila-Poveda et al. 2021) are displayed as pentagons on top of bars. Note that when all GDSs or both reproductive periods co-occur, it is indicative of a reproductive cycle, and pentagons are fully shaded: March and July in females, none in males). At the population level (both sexes in **C**), these reproductive cycles occur relatively every four months and are marked with an asterisk near each shaded pentagon.

The frequency of GDSs varied between sexes (Fig. 6A for females, Fig. 6B for males). Both the Rapid readiness period (Rrp) and the Reproductive achievement period (Rap) were observed year-round in both sexes, with Rrp being more frequent in females than in males. At the population level (combined sexes, Fig. 6C), *Radsia goodallii* exhibited three reproductive cycles, each characterized by the simultaneous presence of all GDSs. These cycles occurred approximately every four months (February–March, July and November), corresponding to one reproductive cycle per thermal phase (Fig. 6C).

Monthly GDS frequencies revealed overall similar patterns between sexes. The most dominant stages were GDS-3 (Ripe), with frequencies ranging from 13 to 82%, and GDS-4 (Spawning), ranging from 15 to 60% (Fig. 6C). GDS-2 (Development) was the third most common stage, occurring year-round in females (10% to 53%), but limited to specific months in males (April–July, September, November, 17% to 50%). GDS-1 (Goniogenesis) and GDS-5 (Resting) were nearly absent in males (Fig. 6B). Interestingly in females, GDS-1 reached a sharply at 60% in February 2018, and GDS-5 was observed in two months of the cooler phase (February and March) and one month of the neutral phase (July), with frequencies ranging from 7 to 20% (Fig. 6A).

### Reproductive season: intensity and duration of the maximum gonad investment (MGI)

**Fig. 7:**
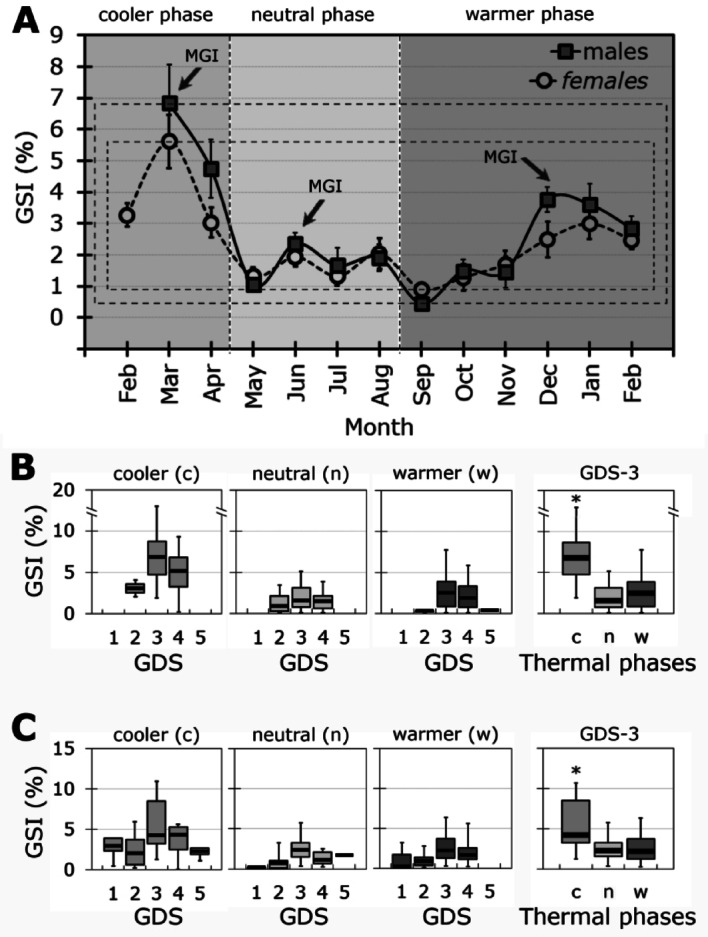
Reproductive Season of *Radsia goodallii* at San Cristóbal Island, Galápagos Archipelago, Ecuador. (**A**) Reproductive intensity (mean GSI% ± SD) and duration for males and females throughout the sampling period. Each thermal phase is highlighted with grey shading. The GSI data are for all sampled individuals at all GDSs (males: n = 158; females: n = 182). Dashed rectangles show the range of the GSI over time for each sex. Note that only two males were collected in February 2018, so they were excluded from this graph. The Maximum Gonad Investment (MGI) refers to the highest GSI% reached; therefore, MGI = GSI_max_ = GDS-3, the Ripe stage of the gonad. Range of GSI values in (**B**) males and (**C**) females for each Gonad Developmental Stage (GDS) and for the GDS-3 Ripe stage per thermal phase. Horizontal lines in (**B**) and (**C**) are medians ± quartile deviation (bold line and box) with min–max ranges (whiskers). Asterisks denote significant differences at *p* < 0.05.

Mean monthly GSI% values ranged from 0.44 ± 0.10 to 6.83 ± 1.24 in males and from 0.89 ± 0.20 to 5.61 ± 0.86 in females (Fig. 7A). Despite the narrower GSI% range in females, no significant differences between sexes were found (F_1, 338_ = 0.6420, *p* = 0.42).

The MGIs (defined as GSI_max_) were found in males and occurred once per thermal phase: in March (cooler phase) with a mean of 6.8%, in June (neutral phase) with a mean of 2.4%, and in December (warmer phase) with a mean of 3.8% (Fig. 7A). Females MGIs were consistently lower than those of males but also peaked once per thermal phase: in March (cooler phase) with a mean of 5.6%, August (neutral phase) with a mean of 2.0%, and January (warmer phase) with a mean of 3.0% (Fig. 7A).

**Fig. 8:**
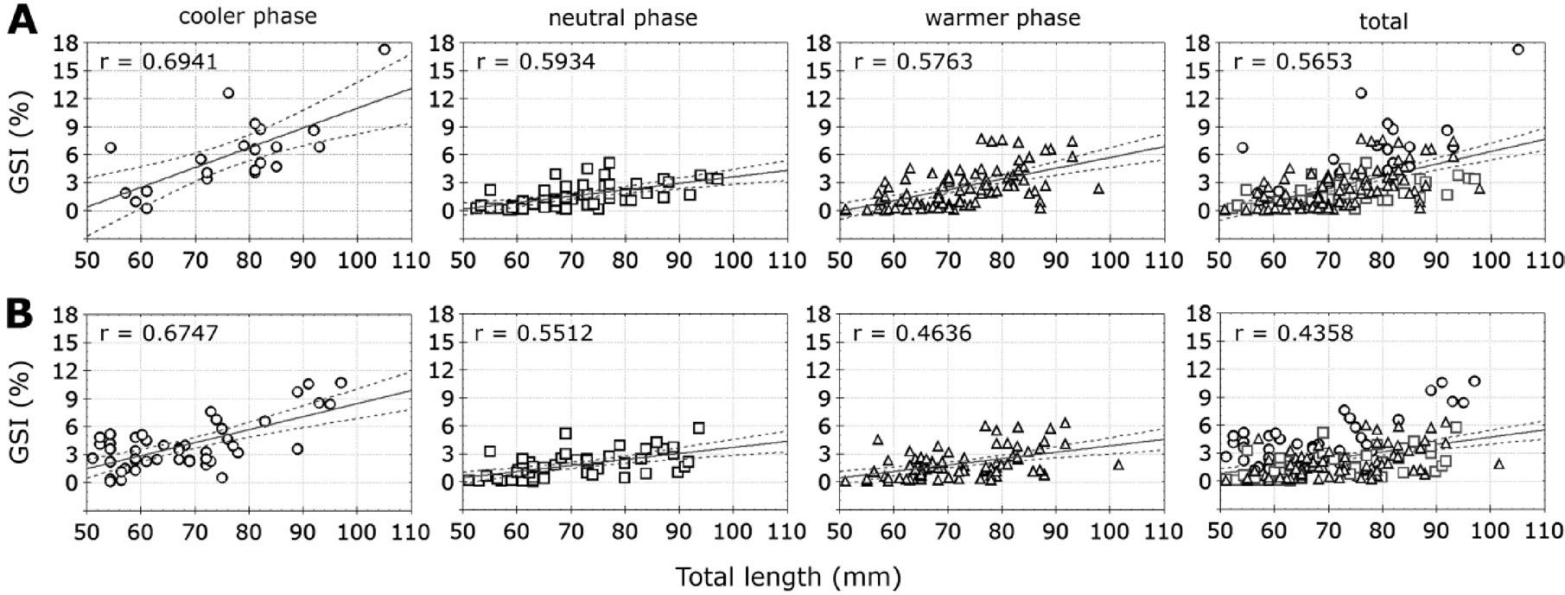
Reproductive Intensity (measured as the Gonadosomatic Index, GSI%) of *Radsia goodallii* in relation to size for (**A**) males and (**B**) females, across thermal phases and in general at San Cristóbal Island, Galápagos Archipelago, Ecuador. Each scatterplot shows a linear fit and regression lines with a confidence level of 0.95.

The duration of MGIs was longest during the warmer phase: two months for males (December and January, *p* > 0.05), and three months for females (December to February, *p* > 0.05) (Fig. 7A). On the other hand, GSI% values did not vary significantly across GDSs within each thermal phase, for sex. However, during the GDS-3 Ripe stage, GSI% was significantly higher in the cooler phase, compared to the neutral and warmer phases in both males and females (Fig. 7B,C). Finally, the reproductive intensity (GSI%) showed a moderate but positive correlation with TL in both sexes (Fig. 8), with the strongest correlation occurring during the cooler phase (Fig. 8).

## Discussion

Our findings indicate that temperature plays a fundamental role in modulating the reproductive traits of *Radsia goodallii*, as an ectothermic species, especially during transitions between thermal phases. These shifts resulted in a rhythmicity in its reproductive cycle of four months. In addition, body size and reproductive intensity were strongly correlated during cooler temperatures. These patterns suggest that this species may adapt its reproductive strategy to align more closely with temperate-zone chitons.

Total Length frequency distributions differed between sexes: skewed to smaller sizes in females, but more uniform sizes in males. Additionally, median TL was significantly smaller in females (68.13 mm) than in males (72.14 mm). To our knowledge, and unexpectedly, this is the first report of sex-related size differences in a chiton species. Male-biased sexual size dimorphism occurs in various taxa; for example, in certain cichlid fish species^86^ and in scorpion flies^87^. For *Radsia goodallii*, males may invest more heavily in somatic growth than females, potentially due to sexual conflict arising from differing reproductive strategies between sexes^88^. Larger body size in males could confer an advantage in sperm competition, as increased size and testes volume are often associated with high sperm production and enhanced fertilization success^89,90^. Sperm competition is a form of intrasexual selection in which the sperm of multiple males compete to fertilize the eggs of a single female^91–93^.

Anthropogenic pressures, such as selective harvesting, can potentially bias population structure^94^, particularly if one sex is disproportionately affected. However, in the case of *Radsia goodallii*, there is no literature available evidencing sexual or spatial segregation, nor of sex-specific behavioral patterns such as differential diurnal/nocturnal activity that might influence exposure to fishing. Moreover, this species does not exhibit external sexual dimorphism, making unlikely sex-selective collection, whether by fishers or researchers. To further minimize sampling bias, individuals were collected randomly, with 17–31 adults sampled monthly. Given the species’ clumped distribution, sampling was limited to a maximum of four individuals per clump to reduce the potential for overharvesting from any single aggregation. Additionally, the observed sex ratio did not deviate significantly from the expected 1:1 male-to-female ratio, further supporting the absence of sex-biased sampling. Having said that, it is recognized that the difference in median TL between sexes (4.0 mm) is relatively small, and further studies, including broader geographic and temporal sampling, experimental validation of reproductive investment, and behavioral assessments, are needed to confirm whether this size disparity reflects a consistent biological pattern or a context-dependent variation.

Interestingly, it was found that size at sexual maturity is similar between sexes, with 69.3 mm for males and 69.5 mm for females. This suggests that females may “delay” reproduction until they reach an optimal size for reproductive success. This means that actively reproducing females in the population had to invest more time and resources in growing first until they reached an optimal size at sexual maturity. However, delayed reproduction is uncommon in invertebrates or species with relatively short life cycles. Nevertheless, to adapt to changing environmental conditions (such as those experienced during thermal phases), plasticity in reproductive timing, or the magnitude of reproductive investment, or a combination of both, may occur. For example^95^, (observed post-threshold plasticity in the insect *Sarcophaga crassipalpis*, where a reproductive delay was triggered as an adaptive response. Conversely, it is also possible that males reproduce earlier (age), since the mean TL value of males was 72.6 mm and the size at sexual maturity was 69.3 mm. However, to determine which reproductive strategy *R. goodallii* follows regarding the speed of male reproductive cycles, future studies should be carried out on this specific aspect.

A statistically significant deviation from the expected 1:1 male-to-female ratio was detected only in February 2018 (Table 1), likely driven by the disproportionately low number of males (n = 2) compared to females (n = 15). While the overall sex ratio did not significantly differ from 1:1 across the study period, females were generally more abundant than males, regardless of thermal phase (Table 1). In chitons, sex ratios are typically close to 1:1. However, when deviations do occur, they are usually male-biased. This has been documented in species such as *Onithochiton lyelli*^57^, *O. neglectus*^96^, and *Chiton pelliserpentis*^65^. In contrast, female-biased sex ratios are less common, though they have been observed in certain species under local mate competition scenarios, where reproductive advantages favor females^61,97,98^. In our study, although only one month showed a statistically significant deviation, a general bias toward females was observed throughout most of the year. This pattern may reflect an unmeasured ecological or physiological factor favoring female abundance in this population. Alternatively, it may represent a natural mechanism of population regulation aimed at ensuring reproductive success.

To further investigate this trend, the sex ratio was considered in combination with the RGES, as both metrics offer insight into reproductive strategy. Notably, females were more abundant than males during most of the study period (Table 1), including two (March and June) of the three months identified as exhibiting MGI (Fig. 7A). Despite this female predominance, the annual RGES was male-biased (Fig. 5), suggesting that males may have increased their reproductive effort—potentially enhancing sperm quantity or quality—to compensate for the numerical dominance of females. Interestingly, broadcast-spawning organisms often exhibit similar GSI values between sexes when sperm competition is high, with little evidence of sexual asymmetry^61,72^. In cases where asymmetry occurs, females typically exhibit higher GSI than males due to the higher energetic cost of producing ova. However, male-biased RGES values have been reported when the energetic investment in sperm remains relatively low, particularly under cooler thermal conditions^71^. Among polyplacophorans, this pattern is more frequently associated with colder environments—either in temperate and subtropical regions or during La Niña phases in tropical zones^66^. Although *Radsia goodallii* is a tropical species, its reproductive pattern may resemble that of temperate species, potentially due to local oceanographic conditions.

When examining the trends in sex ratio and RGES by thermal phase, it was observed that the transition from the cooler to the neutral thermal phase (April–May 2018) coincides with a shift in RGES, resulting in a female-biased gonadal investment (Fig. 5). During this period, females allocated more energy and resources to their gonads, leading to an increase in GSI compared to males. A similar pattern was observed during the transition from the neutral to the warmer thermal phase (August–September 2018, Fig. 5). This shift was more pronounced, with both an increase in RGES values and a longer duration of two months. This behavior might be explained by two scenarios: First, females could increase their GSI as a strategy to ensure successful reproduction in response to changing environmental conditions (thermal phase transitions). According to^72^, higher female GSI typically occurs under conditions of low sperm competition. Second, males might be more adversely affected by these environmental changes, leading to a reduction in their gonadal investment compared to females, which could also result in low sperm competition. In either case, it appears that environmental changes, particularly the transition from neutral to warmer thermal events (such as El Niño) have a significant impact on the reproduction dynamics of this species.

When examining the frequencies of each GDS in *Radsia goodallii* over time, it was observed that during the cooler and neutral thermal phases, females initiate the reproductive cycle in the population, displaying all five GDSs. Males then synchronize with the females, allowing the new reproductive cycle to continue. The GDS-1 Goniogenesis, the GDS-2 Development and GDS-5 Resting stages that comprise the Rrp^79^ appear more frequently and are more prolonged in females than in males. Notably, GDS-1 and GDS-5 are almost absent in males during most months.

Analyzing the Rap, it was found that GDS-3 Ripe and GDS-4 Spawning stages occur throughout the year (except for GDS-3 in February 2018 in females). The duration and frequency of the Rap is more pronounced in males, consistent with findings with *Acanthopleura japonica* (a temperate species in Japan), where males spawned longer than females^99,106^. This suggests that rapid production, development, and release of mature gametes are key components of the reproductive strategy for males in this species. Males exhibit a more prolonged and intense Rap compared to females, particularly during the warmer thermal phase, which also aligns with observations in *Katharina tunicata*^100^ in temperate zones.

Although the reproductive cycle is initiated by females, one factor that appears to trigger spawning in females is the presence of male sperm in the seawater, as males spawn even during months when the frequency of female spawning is less than or equal to 50% of the female population, for example in March, July and December (Fig. 7). In marine invertebrates, males typically spawn a few hours before females^101^, and it is believed that males release substances in their seminal fluid that stimulate nearby females to spawn^102^ or that high sperm density induces spawning in both sexes of the same species^103^. Similar behavior was found in *Chiton articulatus*, where males spawn earlier and more intensively than females in certain months^66^. Overall, there is a general synchrony in the GDSs of females and males; however, this synchrony diminishes during the warmer thermal phase when males are in an intense Rap, while females also have frequent GDS-2 Development stages. This apparent asynchrony was also observed in *Chiton articulatus* during the Godzilla El Niño event (2015–2016)^66^.

Most chiton species exhibit annual reproductive cycles^61,104–106^, However, *Chiton granoradiatus* has been shown to have a semi-annual reproductive cycle^107^. It is well known that the reproductive cycle of marine invertebrates can be influenced by various environmental factors such as water temperature, latitude, salinity, photoperiod, food availability, and thermal phases^78,108–111^, among others. However, the exact mechanisms by which these factors regulate reproductive cycles remain unclear, and variations or interactions among these factors can result in annual, semiannual, or continuous reproductive cycles^112,113^. In the case of *Radsia goodallii*, three distinct reproductive cycles are evident at the population level. The first cycle begins in February–March, the second in July, and the third in November, with each reproductive cycle corresponding to a specific thermal phase and lasting approximately four months. As noted by^110^, in addition to the photoperiod, temperature plays a critical role in coastal invertebrates, where changes in temperature thresholds can trigger physiological responses that are mistimed relative to the calendar date. It is likely that the presence of three thermal phases (cooler, neutral, and warmer), and especially the transitions among them, have contributed to this intra-annual pattern of three reproductive cycles in this species. This suggests that the reproductive response could be species-specific and influenced by evolutionary history or adaptation to the local ecosystem.

At the population level, *Radsia goodallii* exhibits three MGIs throughout the sampling year, and each MGI occurs rhythmically during distinct thermal phases, paralleling the pattern observed in the species reproductive cycle. In the literature, tropical chitons typically show a broad reproductive season, with one or two MGIs spanning several months, usually during the autumn–winter season^114–118^. However, *Radsia goodallii* presents three MGIs, each with varying intensity (%), duration (months) and number of chitons with ripe gonads. The most intense MGI for *R*. *gooadllii* was observed during a short but intense period in February 2018, which coincided with the cooler thermal phase associated with a La Niña event^53^. This thermal anomaly, characterized by strong upwelling and increased nutrient availability enhances marine productivity^44^. Thus, the highest MGI for *R*. *goodallii* occurred during a more productive period than expected for the usual warm, and nutrient-poor season that usually runs from January to May^37,38^. The second MGI, occurring during the neutral thermal phase, showed the lowest intensity and spanned just one month. The third MGI, occurring during the warmer thermal phase, was of intermediate intensity but lasted longer (two months).

When comparing the behavior of *Radsia goodallii* with that of chitons from temperate zones, a marked seasonal trend was observed in both the intensity and duration of their MGIs, with high intensity and limited duration primarily during warmer months^100,106,119^. Similar findings were reported by^120^ in the reproductive cycle of *Argopecten purpuratus*, where a longer MGI duration was observed during El Niño event (1996–1997). While MGI intensity in broadcast spawning invertebrates shows substantial variation depending on locality, latitude and temperature^71,72^, the reproductive pattern of *Radsia goodallii* appears to have responses more like that of temperate zones chitons, with a rhythmic MGIs corresponding to one per each thermal phase. This can be linked to a seasonal response pattern, but one triggered by thermal phases. A similar response was noted by^79^, when comparing the reproductive season of *Chiton articulatus* between pre-ENSO and post ENSO events. Furthermore, in all cases, male MGIs in this study were higher than those of females, particularly during the cooler and warmer thermal phases. Observed this same pattern in a subtropical *Chiton articulatus* population during a La Niña event, concluding that male-biased MGI is more common below average temperatures, either due to La Niña cold thermal phases occurring in the tropics or due to increasing latitude (subtropical and temperate zones)^66^. Therefore, this behavior between the MGI of each sex confirms that *Radsia goodallii* appears to have reproductive responses more like temperate chiton species^66^.

Finally, there are three outcomes when body size is related to reproductive intensity (GSI %) in marine invertebrates: (1) reproductive intensity is not affected by size^60,66^, (2) reproductive intensity increases with increasing size^121^, and (3) reproductive intensity decreases with increasing size due to senescence^122–124^. To explore this effect, the potential impact of body size on reproductive intensity for male and female *Radsia goodallii* during the three thermal phases was assessed. It was found that reproductive intensity moderately increases with size, more notably in males and during the cooler thermal phase. This finding supports the hypothesis that invertebrate species with continuous growth, reproductive output can increase throughout their lifetime until death. This is explained by the physiological theory that once individuals reach maturity, energy initially allocated to somatic growth is gradually redirected to reproduction^125^. This pattern suggests reproductive optimization, particularly in younger adults, likely modulated by the external factors such as temperature or abrupt thermal changes.

## Conclusion

The reproductive cycle and reproductive season of *Radsia goodallii* was characterized by three distinct phases, each aligned with specific thermal conditions, highlighting a unique intra-annual rhythmicity that deviates from the more continuous or single-peak patterns typically seen in tropical species. While the overall sex ratio remained close to 1:1, periodic deviations, coupled with sex-specific variations in gonadal investment, suggest complex reproductive strategies that may be driven by environmental factors and intra-specific competition. The findings underscore the role of thermal variability in shaping reproductive strategies in marine invertebrates, offering crucial insights for future management and conservation efforts for this species.

## Data Availability

The datasets used and/or analysed during the current study available from the corresponding author on reasonable request.
